# Genomic signatures of local directional selection in a high gene flow marine organism; the Atlantic cod (Gadus morhua)

**DOI:** 10.1186/1471-2148-9-276

**Published:** 2009-12-01

**Authors:** Einar E Nielsen, Jakob Hemmer-Hansen, Nina A Poulsen, Volker Loeschcke, Thomas Moen, Torild Johansen, Christian Mittelholzer, Geir-Lasse Taranger, Rob Ogden, Gary R Carvalho

**Affiliations:** 1 National Institute of Aquatic Resources, Technical University of Denmark, Vejlsøvej 39, DK-8600 Silkeborg, Denmark; 2 Department of Biological Sciences, Genetics and Ecology, University of Aarhus, Building 1540, Ny Munkegade, DK-8000 Aarhus C, Denmark; 3 CIGENE, PO Box 5003, N-1434 Ås, Norway; 4 Institute of Marine Research Tromsø, PO Box 6404, N-9294 Tromsø, Norway; 5 Institute of Marine Research, PO Box 1870, Nordnes N-5817 Bergen, Norway; 6 Tepnel Research Products and Services, Appleton Place, Livingston, EH54 7EZ, UK; 7 Molecular Ecology & Fisheries Genetics Laboratory, School of Biological Sciences, Environment Centre Wales, Bangor University, Bangor, Gwynedd LL57 2UW, UK; 8 Current address: University of Basel, Klingelbergstrasse 50/70, CH-4056 Basel, Switzerland

## Abstract

**Background:**

Marine fishes have been shown to display low levels of genetic structuring and associated high levels of gene flow, suggesting shallow evolutionary trajectories and, possibly, limited or lacking adaptive divergence among local populations. We investigated variation in 98 gene-associated single nucleotide polymorphisms (SNPs) for evidence of selection in local populations of Atlantic cod (*Gadus morhua *L.) across the species distribution.

**Results:**

Our global genome scan analysis identified eight outlier gene loci with very high statistical support, likely to be subject to directional selection in local demes, or closely linked to loci under selection. Likewise, on a regional south/north transect of central and eastern Atlantic populations, seven loci displayed strongly elevated levels of genetic differentiation. Selection patterns among populations appeared to be relatively widespread and complex, i.e. outlier loci were generally not only associated with one of a few divergent local populations. Even on a limited geographical scale between the proximate North Sea and Baltic Sea populations four loci displayed evidence of adaptive evolution. Temporal genome scan analysis applied to DNA from archived otoliths from a Faeroese population demonstrated stability of the intra-population variation over 24 years. An exploratory landscape genetic analysis was used to elucidate potential effects of the most likely environmental factors responsible for the signatures of local adaptation. We found that genetic variation at several of the outlier loci was better correlated with temperature and/or salinity conditions at spawning grounds at spawning time than with geographic distance *per se*.

**Conclusion:**

These findings illustrate that adaptive population divergence may indeed be prevalent despite seemingly high levels of gene flow, as found in most marine fishes. Thus, results have important implications for our understanding of the interplay of evolutionary forces in general, and for the conservation of marine biodiversity under rapidly increasing evolutionary pressure from climate and fisheries induced changes in local environments.

## Background

When genotype by environment interactions take place, populations can evolve traits that confer a Darwinian fitness advantage in their local habitat. The process and the resulting patterns are termed "local adaptation" [1]. For adaptive divergence of populations to take place, the evolutionary force of directional selection should be stronger than random genetic drift and the homogenising effect of migration among populations [2]. Accordingly, local adaptations are expected to be rare or absent in species with extensive gene flow, such as many marine fishes [3]. Even though some groups of marine fishes, such as coral reef fishes, have been found to be highly genetically structured [4], more than 40 years of population genetic research has typically demonstrated low levels of genetic differentiation among local demes compared to other fish species and terrestrial organisms [5].

It is notoriously difficult to separate genetic from environmental effects when traits of presumed adaptive value are measured directly in the wild [6]. Furthermore, disentangling genetic from environmental effects on phenotypes is a challenge in relatively large aquatic organisms such as many marine fish species, where experimental designs such as common garden and reciprocal transplantation require large facilities. Alternatively, genome scan approaches using a high number of genetic markers in natural populations can provide a powerful shortcut for demonstrating local selection pressures by allowing the identification of outlier loci with divergent levels of genetic differentiation [7,8]. In particular, targeted approaches investigating gene-associated rather than random DNA marker variation holds great promise [9]. Targeted genome scans have recently been used in natural populations of various organisms such as trees (white spruce) [10] and fish (sticklebacks) [11].

The Atlantic cod (*Gadus morhua *L.) has been the subject of numerous population genetic studies and thus represents one of the best studied marine fish species within the field. Population genetic studies have demonstrated low, albeit significant genetic differentiation on various hierarchical levels, ranging from clear transatlantic differences [12-14] to microgeographical population structure of demes separated by a few tens of kilometres [15-17]. At the same time there is growing evidence that commonly observed variation in life history traits among putative natural populations of Atlantic cod is at least partly determined by genetic rather than environmental differences, see [18] and references therein. Earlier studies have indicated that functional genes may be under selection in natural populations of Atlantic cod. Already in the 1960's the classical studies of haemoglobin polymorphisms in cod by Sick [19] identified a locus (*Hb I*) most likely under environmental selection. Recently, Andersen *et al*. [20] identified the molecular basis as well as the different oxygen binding properties of the haemoglobin alleles, thereby providing the important link between genotypes and phenotypes of adaptive importance. Another example is the identification of the *Pantophysin *(*Pan I*) locus in cod [21] which has been shown to display markedly elevated levels of genetic differentiation compared to presumed neutral markers, patterns which are potentially driven by temperature selection [22,23]. A more extensive survey of genes was recently conducted by Moen *et al*. [24], who reported on the first genome scan approach in the species. They assessed variation in 318 Single nucleotide polymorphisms (SNPs) in a selection of samples from two cod populations (northeast Arctic and Norwegian coastal cod) and identified a number of outlier loci as candidates for being under adaptive evolution. However, in summation, these studies have focused on single genes and/or a restricted geographical area not using random population samples. Consequently, even though Atlantic cod represents one of the most frequently targeted marine species in population genetic research, the genomic architecture as well as the geographical scale and distribution of adaptive divergence in this and other high gene flow marine fishes is still relatively poorly known (see also review by Nielsen *et al*. [25]).

In this study, we tested the hypothesis that populations of Atlantic cod are adapted to local environmental conditions and therefore display genomic signatures of divergent selection. We applied genome scan methods to identify outlier loci potentially associated with adaptive population divergence on global, regional and local scales as well as an explorative landscape genetic analysis focusing on environmental factors suspected to be involved in local adaptation.

## Results

### Genome scan approach

**Figure 1:**
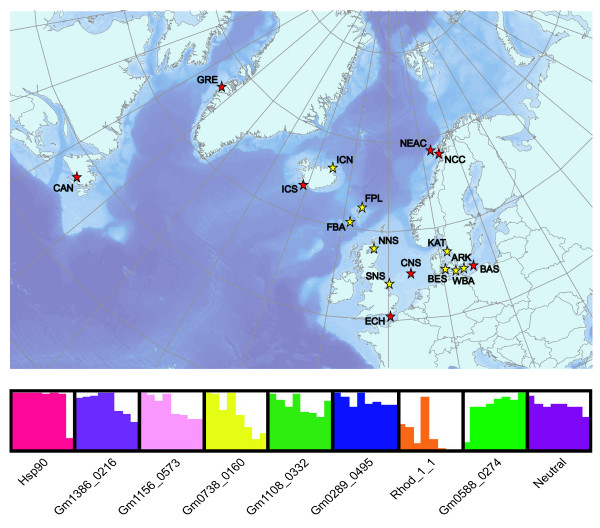
**Map of sampling locations**. Samples were collected from the western Atlantic (CAN), Greenland (GRE), northern Iceland (ICN), southern Iceland (ICS), the Faeroe Bank (FBA), the Faeroe Plateau (FPL), Norwegian coastal cod (NCC), Northeast Arctic cod (NEAC), northern North Sea (NNS), the English Channel (ECH), southern North Sea (SNS), central North Sea (CNS), Kattegat (KAT), Belt Sea (BES), western Baltic Sea (WBA), Arkona Basin (ARK) and the central Baltic Sea (BAS). Red stars indicate samples used in the global analyses, while yellow stars mark additional samples used in regional comparisons (see also Table 1). A bar chart shows allele frequencies in each of the eight samples used in the global analyses. Presented are global outlier loci identified by BAYESCAN along with a representative neutral locus, displaying a global F_ST _corresponding to the global neutral F_ST _(eight outlier loci removed, see Figure 2a)). Populations from left to right for each locus are CAN, GRE, ICS, NEAC, NCC, CNS, ECH, BAS.

**Figure 2:**
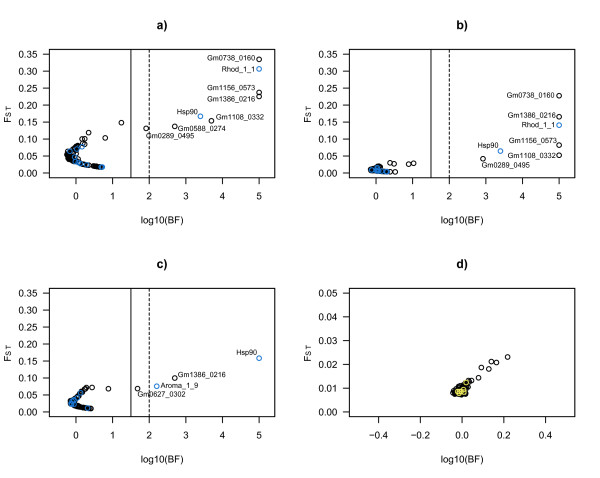
**Spatial and temporal scans for identification of F_ST _outlier loci potentially subject to differential selection**. a) Global dataset with eight samples, b) South-north transect in the Atlantic, c) North Sea - Baltic Sea transect and d) Temporal samples from the Faeroe Bank (note different scale on secondary axis). Shown are Log transformed Bayes factors and locus specific F_ST _from BAYESCAN. Vertical lines mark Log10(BF) of 1.5 (solid) and 2 (broken) corresponding to posterior probabilities of locus effects of 0.97 and 0.99, respectively. Loci with a posterior probability of 1 were ascribed a Log10(BF) of 5. Novel SNPs from this study, which were ascertained in a different set of individuals than used for the random set of markers, are labelled blue in a), b) and c). Loci identified as outliers in the global data set are labelled yellow in d). Monomorphic loci were removed in each simulation.

First, we scanned for global outlier loci by including a selection of major population groups from the western Atlantic (Canada), Greenland, Iceland, northeast Arctic cod, Norwegian coastal cod, North Sea, English Channel and the Baltic Sea (see Table 1 and Figure 1). Eight gene associated SNP loci displayed statistically significant patterns of divergent genetic differentiation consistent with a model of directional selection at the associated gene or other closely linked genes (Figure 2a). Bayes factors for global outliers were high, with seven loci having log10 values above 2 (decisive) and four of these had a log10 Bayes factor of 5, which corresponds to a posterior probability of one. The outliers displayed large differences in allele frequencies among major cod populations (Figure 1). There was no clear trend that particularly divergent allele frequencies (or pairwise F_ST _values, see additional file 1) for outlier loci were associated with one or two specific populations except for Hsp 90 (Baltic) and Gmo0588_0274 (western Atlantic). The candidate loci identified from a wide geographical ascertainment sample in this study (see Methods section) did not show any general trend of decreased or inflated levels of genetic differentiation (Figure 2) compared to the loci identified in a single population ascertainment sample by Moen *et al *[24]. To investigate patterns of selection on a regional scale, we scanned eastern and central Atlantic populations in a south-north transect using samples from the English Channel, southern North Sea, central North Sea, northern North Sea, Faeroe Bank, Faeroe Plateau, Norwegian coastal cod, northeast Arctic cod, Iceland south, Iceland north and Greenland. At this geographical level, seven outlier loci were identified (Figure 2b), all with Bayes factor values well above 2 (log10). The loci identified as likely subjects of directional selection were identical to the loci identified on a global scale, except for the locus Gmo0588_0274, which was not an outlier at this geographical scale. Likewise, divergent allele frequencies (Figure 1) at this locus were only found for the western Atlantic sample (CAN). When leaving out the northeast Arctic cod sample, which generally appeared most genetically divergent, five of the seven outlier loci still remained highly significant (results not shown). Thus, also on this geographical scale, outliers were generally not associated with any particular population. On a local scale, we scanned the geographical proximate but environmentally distant [26] North Sea and the Baltic Sea populations including samples from the transition area. Four outliers, of which three had log10 Bayes factors above 2, signalled evidence of directional selection at this small geographical scale (Figure 2c). Two of the four loci identified here were not identified as outliers on larger geographical scales. Accordingly, the spatial genome scan approach identified overall ten loci where a model of selection was much more likely than a neutral model. A temporal analysis of the Faeroe Bank population using DNA from historical otolith samples collected in 1978 and contemporary tissue samples showed no evidence of directional (or stabilising) selection over time (Figure 2d). Loci identified as likely subjects to directional selection through the spatial analyses (Figure 2a-c) did generally not display patterns of elevated levels of genetic differentiation over time (Figure 2d), which is consistent with temporal stability of spatial directional selection patterns. A significantly higher proportion of the candidate loci (5 of 15) compared to random loci (5 of 83) were identified as outliers (chi-square, P < 0.01, see also additional file 2).

**Table 1:** Atlantic cod samples analysed in the present study.

geographical locality	position	sampling year (abbreviation)	sample size	peak spawning
Baltic Sea^1,3^	54.51° N 15.28° E	1996 (BAS)	40	June
Arkona Basin^3^	54.53° N 13.33° E	1996 (ARK)	40	May
Western Baltic Sea^3^	54.56° N 12.28° E	2007 (WBA)	36	March
Belt Sea^3^	55.11° N 10.28° E	1996 (BES)	40	March
Kattegat^3^	57.15° N 11.35° E	1996 (KAT)	40	April
Central North Sea^1,2,3^	55.17° N 03.39° E	1996 (CNS)	40	April
Southern North Sea^2^	54.29° N 0.02° E	2006 (SNS)	40	April
English Channel^1,2^	50.47° N 0.29° E	2005 (ECH)	40	February
Northern North Sea^2^	58.00° N 03.00° W	2003 (NNS)	40	April
Northeast Arctic cod, Lofoten^1,2^	68.35° N 12.13° E	2003 (NEAC)	40	April
Norwegian coastal cod, Lofoten^1,2^	68.12° N 14.44° E	2003 (NCC)	40	April
Faeroe Plateau^2^	62.53° N 06.18° W	2002 (FPL)	40	March
Faeroe Bank^4^	Unknown	1978 (FBA78)	32	March
Faeroe Bank^2,4^	60.56° N 08.52° W	2002 (FBA)	40	March
Iceland South^1,2^	63.49° N 21.05° W	2002 (ICS)	40	April
Iceland North^2^	66.17° N 15.45° W	2002 (ICN)	40	April
Greenland^1,2^	66.35° N 53.32° W	2005 (GRE)	40	April
Western Atlantic (Canada)^1^	47.39° N 55.24° W	1998 (CAN)	40	May

1) Samples included in global analysis, 2) samples in south-north transect, 3) samples in North Sea-Baltic Sea transect, 4) samples in temporal comparison.

### Landscape genetics

**Figure 3:**
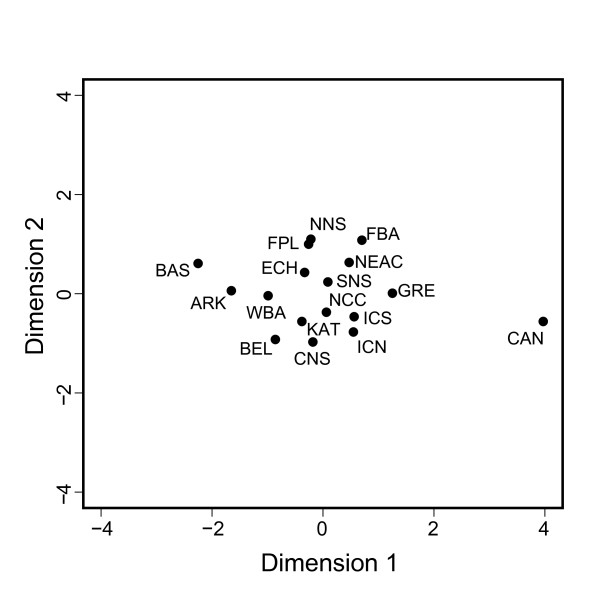
**Multi dimensional scaling plot of "neutral" F_ST _estimates between all spatial samples**. F_ST _estimates are based on all loci, except ten loci identified as potentially under selection (see Figure 2).

The MDS plot (Figure 3) based on all 88 "neutral" loci (all loci except the ten outliers) showed a clear isolation of the western Atlantic sample from Canada (CAN). In addition, the Baltic Sea samples (BAS and ARK) were separated from the remaining central and eastern Atlantic samples (see also additional file 1). The landscape genetic analysis of the global outlier loci revealed that seven of eight loci were significantly associated with temperature on spawning grounds at spawning time (Table 2). Five loci were associated with salinity, while four loci were associated with each of the geographic variables latitude and longitude. In addition, the landscape genetic method also identified associations with environmental variables for loci which had not been identified as outliers using the genome scan method (see additional file 3).

**Table 2:** Significant associations between candidate loci for local adaptation and environmental variables.

	latitude	longitude	temperature	salinity
Rhod_1_1	***	***	***	*
Gm0738_0160	***	***	***	**
Gm1156_0573		***	***	**
Gm1386_0216	***		***	***
Gm0588_0274	***	***	***	
Hsp90				***
Gm0289_0495			**	

Only global F_ST _outliers identified by BAYESCAN are shown (see additional file 3 for more loci and environmental variables in an extended analysis). *** refers to P < 0.001, ** to P < 0.01 and * to P < 0.05 after Bonferroni correction. Hsp90 = *Heat shock protein 90*, Rhod_1_1 = *Rhodopsin*.

## Discussion

This study suggests that loci subject to directional selection can be found on various geographical scales in Atlantic cod. In addition, the results suggest that a large number of loci could be subject to directional selection on a genomic scale among local populations. Local directional selection appears to be general and relatively widespread and can be found on a number of geographical scales since the global or regional outliers identified are not exclusively dependent on one or a few particularly divergent cod populations. Furthermore, although it is difficult to completely disentangle the effects of geographic and environmental distance, a higher proportion of the identified outlier loci were associated with environmental parameters on spawning grounds than with geographic variables. This suggests, but does not establish, these environmental factors as potentially responsible for adaptive divergence among cod populations.

The remaining "neutral" SNP markers generally exhibited very low levels of genetic differentiation and, accordingly, limited resolution of patterns of population relationships, except for the highly divergent western Atlantic and Baltic Sea cod populations. This is somewhat in contrast with evidence from microsatellite markers (e.g. see [12,14,27]), showing higher levels of differentiation but also high variance among loci. Although great care should be taken when comparing levels of genetic structure between markers with different mutational properties [28,29], this difference may suggest that estimates of genetic differentiation from a limited number of microsatellite loci may be even more inflated by hitch-hiking selection than previously assumed. Alternatively, levels of SNP differentiation may be depressed due to the presence of SNPs, which are monomorphic in a number of population samples. This in turn may also explain why we find no evidence for balancing selection in cod. Simulations have shown that scenarios with low levels of population structure provide very low power for detecting loci under balancing selection [30].

The apparent high number of loci with strong statistical support for selection may be somewhat unexpected given the very low level of neutral genetic differentiation among cod populations. However, neutral divergence has in general been shown to be a poor predictor of adaptive trait variation [31]. The proportion of loci subject to selection resembles the findings using genome scan methods for highly structured Atlantic salmon (*Salmo salar*) populations [32]. The result that the North Sea/Baltic Sea cod population comparison only yielded four outliers likely to be subject to directional selection may be surprising given the unique low saline, low oxygen environment for cod in the Baltic Sea. However, although adaptations in Baltic Sea cod may be essential, they may be distributed over few genes, also considering the relatively short history of the Baltic Sea [26]. Alternatively, we cannot exclude that ascertainment bias might have affected results of analyses including Baltic Sea samples (see also below). More detailed studies of the genetic architecture of adaptive traits in Baltic Sea cod are warranted to elucidate these issues. It should be stressed, however, that results from this study should not be taken as a direct estimate of the proportion of genes under divergent (and balancing) selection in different cod populations. Ascertainment bias [33] could potentially have affected the geographical distribution of outliers, since the majority of loci were ascertained from EST libraries consisting of only Norwegian coastal cod [24] and the candidate loci originate from a different and wider ascertainment sample. This could potentially result in higher variability, different levels of genetic differentiation and a different frequency of outliers for comparisons including Norwegian coastal cod and/or our candidate loci. However, marine fishes have been shown to display relatively shallow genetic structure compared to other organisms including other fish species [5], suggesting that ascertainment bias may be less of a problem here [34]. This theoretical consideration is supported by the fact that estimates of heterozygosity and proportion of monomorphic loci were not markedly different in the sample of Norwegian coastal cod (see additional file 2) compared to samples from other populations, except for the most differentiated populations of Canadian cod and Baltic Sea cod. This phenomenon of shared polymorphisms could be caused by the fact that Norwegian coastal cod is closely related to the ancestral population of cod [35] and therefore serve as a superior ascertainment sample (see [36] for an example for *Drosophila*). Furthermore, overall genetic differentiation of "neutral" loci for the EST derived loci and our own candidate loci was almost identical (illustrated in Figure 2), suggesting limited systematic difference between loci from the two ascertainment panels. In conclusion, we only think that ascertainment bias is likely to have significantly reduced the identified number of loci under selection for the very divergent Canadian cod. However, even if the proportion of outliers in the most divergent populations within the remaining cluster (i.e. northeast Arctic cod and Baltic Sea cod) might be mildly underestimated, we expect ascertainment bias to play a relatively minor role on regional and local scales based on theoretical expectations and our own evaluation of data.

Another potential concern in relation to establishing outliers as products of directional selection is the high number of loci tested (98). With an alpha level of 0.05 we would expect five significant outliers, which in fact is not so far from the present results. However, the statistical support for individual outliers identified here is generally much stronger. We should expect only one outlier by chance with a Bayes factors above 2 (log10), but the computed values are almost exclusively above 2 (log10), which is generally interpreted as "decisive". Four loci have log10 values as high as 5, which was the value ascribed to posterior probabilities of 1 (Bayes factor is infinity). Accordingly, we are still convinced that the majority of these outliers are affected by directional selection and not just outliers by chance. We think that our evidence for selection is as strong as possibly achievable given the statistical method and the number of loci at hand.

As evidenced above, the identified outlier genes originate both from randomly chosen gene associated SNPs and from specifically targeted SNPs in candidate genes of known functions. Interestingly, a relatively high proportion of candidate genes showed evidence of being subject to selection compared to the randomly chosen SNPs (app. 30% versus app. 6%). The identified outlier genes code for proteins with highly divergent functions (additional file 4), suggesting that directional selection for local adaptations in cod occur along multiple environmental dimensions. For example, heat-shock protein genes, represented by *Hsp90*, have been shown to be involved in various stress responses in fish including temperature, salinity and pollution [37]. *Aromatase *plays a regulatory role in sex determination, gametogenesis, central nervous system development and sex behaviour [38] and *rhodopsin *is a pigment gene involved in the formation of photoreceptor cells and the perception of light [39]. Alternatively, since temperature is important for proper functioning of all proteins and physiological processes [40,41] temperature differences among geographical locations alone could be driving the evolutionary response for all genes in concert. The landscape genetic analysis partly supports this hypothesis, since almost all outlier loci appear to have alleles significantly associated with temperature. However, salinity also emerges as a likely driver of directional selection for local adaptation in Atlantic cod. Both temperature and salinity have been associated with phenotypic variation in cod. For instance, body shape variation was observed among juvenile cod originating from different Canadian spawning populations when reared under different temperature conditions [42]. Likewise, adaptive trait variation in response to salinity has been strongly suggested for cod in the Baltic Sea, showing divergent egg buoyancy and sperm mobility [43]. Still, the landscape genetic analysis should only be considered explorative. We have investigated a small subset of environmental factors potentially affecting allele frequencies at outliers. Likewise, most parameters are correlated to some extent, leading to significant associations with both geography and environment. This is illustrated by the positive correlations with environment for a number of genes not identified as outliers. It is not a simple task to unambiguously establish the link between genomic signatures of local adaptation and specific environmental conditions, particularly if only correlation analyses are applied. More research is needed on cod reared under controlled conditions in order to establish the genetic architecture of traits subject to local directional selection leading to fitness difference between native and non-native fish [2].

## Conclusion

Previously, genetic evidence of local adaptation at the DNA level in marine fishes has been inferred from single genes [20,44,45], or from restricted geographical areas [24]. To our knowledge, the present targeted genome scan approach represents the first attempt to elucidate genomic signatures of directional selection in natural populations of a marine fish on various geographical scales across its range. Even though our understanding of the genetic architecture of adaptive evolution in marine fishes is not as advanced as in other well studied fish species (e.g. sticklebacks [46] or whitefish [47]), our findings strongly suggest that marine fishes are not only isolated into local populations, even on relatively small geographical scales [4]. Despite being connected by variable levels of gene flow, these populations can indeed follow semi-independent adaptive evolutionary trajectories shaped by selection by their local environments. Thus, adaptive population divergence seems to be possible and may even be prevalent despite seemingly high levels of gene flow often found in marine fishes. Our findings have implications for sustainable management of marine fishes by underpinning the local population as the focal management unit. Thus, we cannot rely on immigration to rehabilitate declining populations, since non-native individuals will have lower fitness and therefore are less likely to be successful in their new environment. Moreover, the extirpation of local populations may represent irreversible changes to the gene pool and associated adaptive evolutionary potential [48]. Recent assertions of global warming causing recruitment failure in cold-adapted North Sea cod emphasize the importance of maintaining the adaptive potential of exploited species [49]. Likewise, predictions on future distribution and abundance of marine fishes should not rely on simplified spatial models viewing whole species as single units with global bioclimatic niches. In order to improve the predictive power of future responses to environmental change, information on the dynamics of locally adapted populations, as well as on population-specific adaptive trait values, will be required.

## Methods

### Sampling

In total 708 adult individuals were analysed from 17 spatial samples and one temporal sample, all consisting of approximately 40 individuals (Table 1 and Figure 1). Spatial analyses were conducted using samples from three different hierarchical levels; global (CAN, GRE, ICS, NCC, NEAC, ECH, CNS and BAS), a regional south-north transect of central and north-eastern Atlantic population samples (ECH, SNC, CNS, NNS, FBA, FPL, NCC NEAC, ICS, ICN and GRE) and a North Sea - Baltic Sea transect (CNS, KAT, BES, WBA, ARK and BAS). The temporal comparison was conducted on samples from the Faeroe Bank in the eastern Atlantic (FBA78 and FBA).

### DNA analysis

DNA was extracted from tissue or archived otoliths (FBA78) using the proteinase K/chelex method [50]. Overall 98 SNPs were genotyped by the MassARRAY system from SEQUENOM. Eighty-six SNPs were selected from previously published EST derived SNPs from cDNA libraries of Norwegian coastal cod (ascertainment sample) [24]. Of these, eighty were randomly selected, while six were specifically selected because they were expected to play a role in local adaptation. In addition, we analysed twelve novel SNPs developed for this study (Table 3 and additional file 5). These SNPs were discovered by screening genomic DNA with primers designed from public teleostei or Atlantic cod sequences. Individuals for the initial screen (ascertainment sample) were chosen in order to cover the major part of the distributional area of Atlantic cod (CAN, NEAC, CNS and BAS). Thus, among the total of 98 SNPs, fifteen were from candidate genes for adaptive evolution, with functions primarily related to temperature stress, growth and reproduction (see additional file 2).

**Table 3:** Novel SNPs analysed in this study

locus name	protein	snp location	**genbank accession no**.
Aroma_1_9	Aromatase	Intronic	DQ402370
Aroma_2_3	Aromatase	Exonic	DQ402370
FshB_1_1	Follicle stimulating hormone	Exonic	DQ402373
Gh_1_1	Growth hormone	Intronic	EU676171
Haemoglobin_alpha	Haemoglobin alpha subunit	Intronic	FJ666966
Haemoglobin_beta	Haemoglobin beta subunit	Intronic	FJ666984
Hsp90	Heat shock protein 90	Intronic	GU063866
LDHB#1	Lactate Dehydrogenase B	Intronic	AJ609233
Rhod_1_1	Rhodopsin	Exonic	AF385832
S2_1_1	Ribosomal protein S2	Intronic	AY292468
S2_1_6	Ribosomal protein S2	Intronic	AY292468
Gm_snp1	Unknown	Unknown	GU063867

GenBank accession numbers refer to reference sequences for each locus. See additional file 5 for detailed information on individual SNPs.

### Statistical analysis

Observed and expected levels of heterozygosity and tests for conformance to Hardy-Weinberg Equilibrium following [51] were calculated for each locus and population in ARLEQUIN ver. 3.1 [52]. Summary statistics for each locus are presented in additional file 2. Overall and locus-specific F_ST _values were estimated by Weir and Cockerham's θ [53] in GENEPOP 4.0.10 [54]. Pairwise single locus F_ST _between samples used in the global analyses were estimated for each of the loci identified as outliers in BAYESCAN. In addition, pairwise multi-locus F_ST _were estimated for all loci, excluding the outlier loci (see additional file 1). Likewise, multi-locus "neutral" pairwise F_ST _were estimated between all samples and used to generate a MDS plot (Figure 3) using the program Vista 5.6.3. [55].

To detect signatures of natural selection we used the Bayesian likelihood method implemented via reversible jump Markov Chain Monte Carlo in BAYESCAN [30]. The Bayesian regression approach implemented in BAYESCAN has several advantages over the widely used approach based on summary statistics implemented in FDIST2 [56]. Firstly, in BAYESCAN F_ST _is modelled using a logistic regression model implementing a locus effect and a population effect, while simulations in FDIST2 are based on an Island Model. Thus, BAYESCAN allow population specific F_ST _in contrast to the symmetrical Island Model in FDIST2, and therefore BAYESCAN should be appropriate for Atlantic cod, which is expected to display non-symmetrical patterns of gene flow among natural populations. Secondly, and supporting this theoretical reasoning, analyses of simulated data have shown that the Bayesian regression approach performs slightly better than FDIST2 when scenarios deviate from the standard Island Model [56,57]. Thirdly, FDIST2 simulates a distribution of F_ST _from the empirical estimate, which is itself influenced by loci potentially under selection. This problem can be alleviated by iteratively removing outlier loci before running the programme [30,56]. However, this approach is subject to a certain degree of subjectivity, which is avoided in the Bayesian approach in BAYESCAN. BAYESCAN directly estimates the posterior probability that a locus is under selection (see below) in contrast to an earlier method build on the same basic regression model, which provided an approximated p-value [57]. In short, BAYESCAN estimates the probability that a locus is under selection by calculating a Bayes factor, which is simply the ratio of the posterior probabilities of two models (selection/neutral) given the data. A Bayes factor between 32 and 100 (log10 = 1.5 - 2) is "very strong evidence" of different statistical support for the two models and corresponds to a posterior probability between 0.97-0.99. For Bayes factors above 100 (log10 > 2) the evidence is interpreted as "decisive" and correspond to posterior probabilities between0.99 and 1.

In BAYESCAN, monomorphic loci were removed from each run. Following 10 pilot runs of 5000 iterations and an additional burn-in of 50000 iterations, we used 100000 iterations (sample size of 5000 and thinning interval of 20) to identify loci under selection from locus specific Bayes factors. A Bayes factor of infinity, corresponding to a posterior probability of 1, was assigned a log10 value of 5. In order to alleviate potential problems arising from allele frequency correlations between samples [58,59], we ran a global analysis with eight representative samples covering the distributional area of the species rather than including all available samples. In addition, we ran regional analyses with more samples from restricted geographical areas.

A spatial analysis, using the program SAM [60], was performed in order to identify associations between alleles and environmental variables. Two environmental parameters, temperature and salinity, as well as latitude and longitude were assessed (see below). We chose to use temperature and salinity at spawning time (but see also additional file 3 for analyses applying a wider set of closely related environmental variables) since this is as close as possible to the most critical time of the cod life-cycle, i.e. when the life stages (i.e. egg and larvae) have little option for actively avoiding unfavourable conditions (see also discussion in [61]). Since the early life stages of Atlantic cod are pelagic, we would expect a relatively strong correlation between surface/near surface data and the actual environmental conditions experienced by these life stages at most sampling locations. Temperature data were obtained from NOAAs Optimal Interpolation version 2 monthly SST analyses (OI.v2 monthly Sea Surface Temperature, available from the IRI/LDEO Climate Data Library at http://ingrid.ldgo.columbia.edu). Salinity data were collected from NOAAs Global Ocean Data Assimilation System (GODAS monthly below sea level salinity at a depth of 5 m, http://ingrid.ldgo.columbia.edu) and from the oceanographic database managed by the International Council for the Exploration of the Sea (ICES surface salinity data, available from http://www.ices.dk). Environmental data were integrated in a Geographic Information System (ArcGIS 9 from ESRI) in order to extract data covering a latitude/longitude grid of 0.5° *0.5° to 1° *1° around each sample position. Environmental variables used in SAM were means of a sampling period from 1990 to 2007. The null hypothesis of no association between an allele and an environmental variable was rejected if the examined variable explained the observed allelic distribution better than a model with a constant only. We changed the critical P value from 0.05 to 0.01 and 0.001 for the implemented Wald test to demonstrate robustness of significant associations. Since only two alleles were present at each locus, leaving only one independent variable, we present the results as associations between locus and environmental variable.

## Authors' contributions

EN conceived and designed the study and drafted the manuscript. JH-H analysed the majority of the data, participated in the design of the study as well as drafting and commenting on the manuscript. NAP participated in the design of the study, data analysis and commented on previous drafts of the manuscript. VL participated in the design of the study and commented on manuscript drafts. TM, TJ, CM, G-LT and RO provided data and commented on manuscript drafts. GC participated in the design of the study and in drafting and commenting of the manuscript. All authors have read and approved the final version of the manuscript.

## Supplementary Material

Additional file 1 **Pairwise F_ST_**. Pairwise Fst for eight samples used in the global analysis (See Figure 1 and Table 1). Shown are single locus estimates for each of the ten loci identified as candidates for adaptive evolution (see Figure 2) and multi-locus estimates based on all loci, excluding the ten outliers. Click here for file

Additional file 2 **Summary statistics**. Observed and expected levels of heterozygosity and tests for conformance to Hardy-Weinberg Equilibrium per locus and sample. Click here for file

Additional file 3 **Results from extended spatial analyses**. All loci identified by SAM [60]to be significantly associated with one or more of nine variables (seven environmental variables, latitude and longitude) in global and regional analyses. Click here for file

Additional file 4 **Putative physiological function of outlier loci**. Blast hits for outlier loci were obtained by blasting contig sequences from [24] or own sequences (see Table 3 for reference sequences in GenBank) against the nucleotide database at GenBank. Biological processes and molecular functions were identified in the Gene Ontology database (http://www.geneontology.org, [62]). Where available, gene ontology is reported for *Danio rerio *(^a^), otherwise *Homo sapiens *(^b^) is used as reference. Click here for file

Additional file 5 **Novel SNPs analysed in this study**. New SNPs analysed in this study with PCR primers to amplify a fragment containing the SNP. Click here for file
