# Supplementary material for: Genomic signatures of local directional selection in a high gene flow marine organism; the Atlantic cod (Gadus morhua)
Source: BMC Evol Biol. 2009 Dec 1;9:276. doi: 10.1186/1471-2148-9-276 (PMC2790465; doi:10.1186/1471-2148-9-276)
Supplement: Additional file 1 — Pairwise FST. Pairwise Fst for eight samples used in the global analysis (See Figure 1 and Table 1). Shown are single locus estimates for each of the ten loci identified as candidates for adaptive evolution (see Figure 2) and multi-locus estimates based on all loci, excluding the ten outliers. [file 1471-2148-9-276-S1.DOC]

**Additional file 1. Pairwise Fst.**

Pairwise Fst for eight samples used in the global analysis (See Figure 1 and Table 1). Shown are single locus estimates for each of the ten loci identified as candidates for adaptive evolution (see Figure 2) and multi-locus estimates based on all loci, excluding the ten outlier loci.

| **Gm1386_0216** | CAN | GRE | ICS | NEAC | NCC | CNS | ECH | BAS |
| --- | --- | --- | --- | --- | --- | --- | --- | --- |
| CAN |  |  |  |  |  |  |  |  |
| GRE | -0.009 |  |  |  |  |  |  |  |
| ICS | -0.006 | -0.015 |  |  |  |  |  |  |
| NEAC | 0.058 | 0.032 | 0.018 |  |  |  |  |  |
| NCC | 0.058 | 0.032 | 0.018 | -0.013 |  |  |  |  |
| CNS | 0.128 | 0.165 | 0.185 | 0.285 | 0.285 |  |  |  |
| ECH | 0.180 | 0.224 | 0.247 | 0.365 | 0.365 | -0.011 |  |  |
| BAS | 0.326 | 0.365 | 0.390 | 0.483 | 0.483 | 0.058 | 0.036 |  |

| **Gm1108_0332** | CAN | GRE | ICS | NEAC | NCC | CNS | ECH | BAS |
| --- | --- | --- | --- | --- | --- | --- | --- | --- |
| CAN |  |  |  |  |  |  |  |  |
| GRE | 0.059 |  |  |  |  |  |  |  |
| ICS | 0.181 | 0.037 |  |  |  |  |  |  |
| NEAC | -0.013 | 0.057 | 0.178 |  |  |  |  |  |
| NCC | 0.262 | 0.099 | 0.001 | 0.258 |  |  |  |  |
| CNS | 0.291 | 0.118 | 0.007 | 0.287 | -0.013 |  |  |  |
| ECH | 0.367 | 0.190 | 0.050 | 0.363 | 0.008 | -0.003 |  |  |
| BAS | 0.083 | -0.008 | 0.018 | 0.081 | 0.071 | 0.087 | 0.156 |  |

| **Hsp90** | CAN | GRE | ICS | NEAC | NCC | CNS | ECH | BAS |
| --- | --- | --- | --- | --- | --- | --- | --- | --- |
| CAN |  |  |  |  |  |  |  |  |
| GRE | - |  |  |  |  |  |  |  |
| ICS | - | - |  |  |  |  |  |  |
| NEAC | - | - | - |  |  |  |  |  |
| NCC | 0.013 | 0.012 | 0.013 | 0.012 |  |  |  |  |
| CNS | 0.001 | 0.001 | 0.001 | 0.001 | -0.010 |  |  |  |
| ECH | 0.014 | 0.013 | 0.014 | 0.013 | -0.013 | -0.009 |  |  |
| BAS | 0.772 | 0.769 | 0.772 | 0.769 | 0.735 | 0.746 | 0.732 |  |

| **Gm0289_0495** | CAN | GRE | ICS | NEAC | NCC | CNS | ECH | BAS |
| --- | --- | --- | --- | --- | --- | --- | --- | --- |
| CAN |  |  |  |  |  |  |  |  |
| GRE | 0.033 |  |  |  |  |  |  |  |
| ICS | 0.218 | 0.097 |  |  |  |  |  |  |
| NEAC | 0.000 | 0.061 | 0.246 |  |  |  |  |  |
| NCC | 0.163 | 0.051 | -0.007 | 0.192 |  |  |  |  |
| CNS | 0.124 | 0.022 | 0.010 | 0.154 | -0.011 |  |  |  |
| ECH | 0.174 | 0.058 | -0.007 | 0.203 | -0.014 | -0.007 |  |  |
| BAS | 0.173 | 0.059 | -0.007 | 0.201 | -0.013 | -0.006 | -0.012 |  |
|  |  |  |  |  |  |  |  |  |

**Additional file 1 continued**

| **Rhod_1_1** | CAN | GRE | ICS | NEAC | NCC | CNS | ECH | BAS |
| --- | --- | --- | --- | --- | --- | --- | --- | --- |
| CAN |  |  |  |  |  |  |  |  |
| GRE | -0.004 |  |  |  |  |  |  |  |
| ICS | 0.221 | 0.158 |  |  |  |  |  |  |
| NEAC | 0.381 | 0.451 | 0.761 |  |  |  |  |  |
| NCC | 0.130 | 0.077 | 0.004 | 0.678 |  |  |  |  |
| CNS | 0.366 | 0.300 | 0.034 | 0.864 | 0.094 |  |  |  |
| ECH | 0.418 | 0.353 | 0.075 | 0.895 | 0.143 | -0.003 |  |  |
| BAS | 0.468 | 0.406 | 0.133 | 0.924 | 0.201 | 0.040 | 0.013 |  |

| **Gm0588_0274** | CAN | GRE | ICS | NEAC | NCC | CNS | ECH | BAS |
| --- | --- | --- | --- | --- | --- | --- | --- | --- |
| CAN |  |  |  |  |  |  |  |  |
| GRE | 0.502 |  |  |  |  |  |  |  |
| ICS | 0.502 | -0.017 |  |  |  |  |  |  |
| NEAC | 0.590 | 0.000 | -0.001 |  |  |  |  |  |
| NCC | 0.687 | 0.054 | 0.053 | 0.010 |  |  |  |  |
| CNS | 0.733 | 0.097 | 0.095 | 0.040 | -0.004 |  |  |  |
| ECH | 0.671 | 0.042 | 0.040 | 0.003 | -0.010 | 0.001 |  |  |
| BAS | 0.836 | 0.247 | 0.246 | 0.178 | 0.103 | 0.067 | 0.115 |  |

| **Gm1156_0573** | CAN | GRE | ICS | NEAC | NCC | CNS | ECH | BAS |
| --- | --- | --- | --- | --- | --- | --- | --- | --- |
| CAN |  |  |  |  |  |  |  |  |
| GRE | 0.017 |  |  |  |  |  |  |  |
| ICS | 0.124 | 0.044 |  |  |  |  |  |  |
| NEAC | 0.014 | -0.016 | 0.065 |  |  |  |  |  |
| NCC | 0.353 | 0.271 | 0.111 | 0.293 |  |  |  |  |
| CNS | 0.377 | 0.296 | 0.133 | 0.318 | -0.015 |  |  |  |
| ECH | 0.441 | 0.364 | 0.198 | 0.386 | 0.002 | -0.008 |  |  |
| BAS | 0.443 | 0.366 | 0.200 | 0.388 | 0.004 | -0.006 | -0.015 |  |

| **Gm0738_0160** | CAN | GRE | ICS | NEAC | NCC | CNS | ECH | BAS |
| --- | --- | --- | --- | --- | --- | --- | --- | --- |
| CAN |  |  |  |  |  |  |  |  |
| GRE | 0.013 |  |  |  |  |  |  |  |
| ICS | 0.293 | 0.236 |  |  |  |  |  |  |
| NEAC | 0.000 | -0.008 | 0.263 |  |  |  |  |  |
| NCC | 0.381 | 0.326 | 0.005 | 0.353 |  |  |  |  |
| CNS | 0.575 | 0.529 | 0.135 | 0.552 | 0.061 |  |  |  |
| ECH | 0.785 | 0.749 | 0.380 | 0.767 | 0.279 | 0.087 |  |  |
| BAS | 0.697 | 0.657 | 0.268 | 0.677 | 0.174 | 0.022 | 0.009 |  |

**Additional file 1 continued**

| **Aroma_1_9** | CAN | GRE | ICS | NEAC | NCC | CNS | ECH | BAS |
| --- | --- | --- | --- | --- | --- | --- | --- | --- |
| CAN |  |  |  |  |  |  |  |  |
| GRE | -0.001 |  |  |  |  |  |  |  |
| ICS | -0.013 | -0.008 |  |  |  |  |  |  |
| NEAC | -0.013 | -0.009 | -0.015 |  |  |  |  |  |
| NCC | 0.006 | -0.010 | -0.005 | -0.006 |  |  |  |  |
| CNS | -0.006 | -0.010 | -0.012 | -0.012 | -0.010 |  |  |  |
| ECH | -0.001 | -0.012 | -0.010 | -0.011 | -0.014 | -0.013 |  |  |
| BAS | 0.382 | 0.473 | 0.424 | 0.423 | 0.495 | 0.456 | 0.479 |  |

| **Gm0627_0302** | CAN | GRE | ICS | NEAC | NCC | CNS | ECH | BAS |
| --- | --- | --- | --- | --- | --- | --- | --- | --- |
| CAN |  |  |  |  |  |  |  |  |
| GRE | 0.007 |  |  |  |  |  |  |  |
| ICS | 0.057 | 0.002 |  |  |  |  |  |  |
| NEAC | 0.050 | -0.002 | -0.013 |  |  |  |  |  |
| NCC | 0.013 | -0.014 | -0.002 | -0.005 |  |  |  |  |
| CNS | 0.021 | -0.014 | -0.007 | -0.010 | -0.014 |  |  |  |
| ECH | 0.067 | 0.007 | -0.014 | -0.013 | 0.003 | -0.005 |  |  |
| BAS | 0.087 | 0.178 | 0.274 | 0.263 | 0.191 | 0.211 | 0.291 |  |

| **”Neutral” loci** | CAN | GRE | ICS | NEAC | NCC | CNS | ECH | BAS |
| --- | --- | --- | --- | --- | --- | --- | --- | --- |
| CAN |  |  |  |  |  |  |  |  |
| GRE | 0.068 |  |  |  |  |  |  |  |
| ICS | 0.108 | 0.001 |  |  |  |  |  |  |
| NEAC | 0.115 | 0.003 | 0.001 |  |  |  |  |  |
| NCC | 0.116 | 0.007 | 0.002 | 0.006 |  |  |  |  |
| CNS | 0.118 | 0.008 | 0.001 | 0.006 | -0.002 |  |  |  |
| ECH | 0.122 | 0.011 | 0.000 | 0.000 | 0.002 | -0.001 |  |  |
| BAS | 0.201 | 0.059 | 0.050 | 0.042 | 0.042 | 0.039 | 0.036 |  |
