# Supplementary material for: Genomic signatures of local directional selection in a high gene flow marine organism; the Atlantic cod (Gadus morhua)
Source: BMC Evol Biol. 2009 Dec 1;9:276. doi: 10.1186/1471-2148-9-276 (PMC2790465; doi:10.1186/1471-2148-9-276)
Supplement: Additional file 3 — Results from extended spatial analyses. All loci identified by SAM [60]to be significantly associated with one or more of nine variables (seven environmental variables, latitude and longitude) in global and regional analyses. [file 1471-2148-9-276-S3.DOC]

**Additional file 3. Results from extended spatial analyses.**

All loci identified by SAM (Joost *et al.* 2008) to be significantly associated with one or more of nine variables (seven environmental variables, latitude and longitude) in global and regional analyses. *** refers to P<0.001, ** to P<0.01 and * to P<0.05 after Bonferroni corrections.

|  | locus | latitude | longitude | temperature | | | | | salinity | |
| --- | --- | --- | --- | --- | --- | --- | --- | --- | --- | --- |
|  |  |  |  | spawning month | juvenile month | annual variance | annual minimum | annual maximum | spawning month | annual variance |
| Global | Rhod_1_1 | *** | *** | *** | * |  | *** | *** |  | *** |
|  | Gm0738_0160 | *** | *** | *** | *** |  | *** | *** | ** | *** |
|  | Gm1156_0573 |  | *** | *** |  |  | *** | *** | * | *** |
|  | Gm1386_0216 | *** |  | *** | *** | *** |  | *** | *** | *** |
|  | Gm0588_0274 | *** | *** | *** |  | *** | *** |  |  | *** |
|  | Hsp90 |  |  | *** | *** | *** |  | *** | *** | * |
|  | Gm1108_0332 |  |  |  |  |  | ** |  |  |  |
|  | Gm0289_0495 |  |  | * |  |  |  |  |  |  |
|  | Aroma_1_9 |  |  | *** | *** | *** |  | *** | ** |  |
|  | Gm0411_0388 | *** | *** | *** |  | *** | *** |  |  | *** |
|  | Gm0704_0401 | ** | *** | *** |  | *** | *** |  |  | *** |
|  | Gm0916_0170 |  | *** | ** | * |  |  |  | *** |  |
|  | Gm392_1005 |  |  | *** | *** |  |  |  | *** |  |
|  | Aroma_2_3 |  |  |  |  | * | ** |  |  |  |
|  | Gm0627_0302 |  |  |  |  | * |  |  | *** |  |
|  | Gm1330_0731 |  | ** | * |  |  |  |  |  | * |
|  | S2_1_1 |  |  |  |  |  |  |  | * |  |
|  | Gm0250_0086 |  | * | * |  |  |  |  |  |  |
|  | Gm0248_0453 |  | ** | ** |  |  |  |  |  | ** |
|  | Gm1090_0134 |  | *** |  |  |  |  |  |  |  |
|  | Gm0433_0214 |  | *** | * |  |  | ** |  |  | *** |
|  | Gm1253_0140 |  | ** |  |  |  |  |  |  |  |
|  | Gm1152_0344 |  | *** |  |  |  | * |  |  | ** |
|  | Gm0974_0477 |  |  | * |  |  |  |  |  |  |
|  | Gm370_0380 |  |  | ** |  |  |  |  | * |  |
|  | Gm374_0856 |  | *** |  |  |  |  |  |  |  |
|  | Gm155_0155 |  |  |  |  |  |  |  | * |  |
| South - | Rhod_1_1 | *** |  | *** |  |  | *** | *** | *** | *** |
| North | Gm0738_0160 | *** | *** | *** | *** |  | *** | *** | *** | *** |
|  | Gm1156_0573 | * |  |  |  | * |  | * |  |  |
|  | Gm1386_0216 | *** |  | * |  |  | * | *** |  |  |
|  | Hsp90 |  |  |  | * |  |  |  |  |  |
|  | Gm1253_0140 |  |  |  |  | ** |  |  |  |  |
|  | Gm278_0092 |  |  |  |  | *** |  | *** |  |  |
|  | Gm155_0155 |  |  |  |  |  |  | * |  |  |
| North Sea - | Gm1386_0216 |  |  | *** | ** |  |  |  |  |  |
| Baltic Sea | Hsp90 |  | *** | *** | *** | *** | *** | ** | *** | ** |
|  | Aroma_1_9 |  | *** | *** | *** |  |  |  | ** |  |
|  | Gm0627_0302 |  |  | *** | *** |  |  |  |  | * |
|  | Gm370_0380 |  |  | *** | ** |  |  |  |  |  |

Hsp90 = *Heat shock protein 90*, Rhod_1_1 = *Rhodopsin*, Aroma_1_9 = *Aromatase*.

**References**

Joost S, Kalbermatten M, Bonin A: **Spatial analysis method (SAM): a software tool combining molecular and environmental data to identify candidate loci for selection.** *Mol Ecol Res* 2008, **8**: 957-960.
