# Supplementary material for: Genomic signatures of local directional selection in a high gene flow marine organism; the Atlantic cod (Gadus morhua)
Source: BMC Evol Biol. 2009 Dec 1;9:276. doi: 10.1186/1471-2148-9-276 (PMC2790465; doi:10.1186/1471-2148-9-276)
Supplement: Additional file 4 — Putative physiological function of outlier loci. Blast hits for outlier loci were obtained by blasting contig sequences from [24] or own sequences (see Table 3 for reference sequences in GenBank) against the nucleotide database at GenBank. Biological processes and molecular functions were identified in the Gene Ontology database (http://www.geneontology.org, [62]). Where available, gene ontology is reported for Danio rerio (a), otherwise Homo sapiens (b) is used as reference. [file 1471-2148-9-276-S4.DOC]

**Additional file 4. Putative physiological function of outlier loci.**

Blast hits for outlier loci were obtained by blasting contig sequences from Moen *et al.* (2008) or own sequences (see Table 3 for reference sequences in GenBank) against the nucleotide database at GenBank. Biological processes and molecular functions were identified in the Gene Ontology database ([www.geneontology.org](http://www.geneontology.org/), Ashburner *et al.* 2000). Where available, gene ontology is reported for *Danio rerio* (^a^), otherwise *Homo sapiens* (^b^) is used as reference.

| locus | outlier | gene/blast hit | biological processes | molecular functions |
| --- | --- | --- | --- | --- |
| Gm0289_0495 | Global and regional | NA |  |  |
| Gm0588_0274 | Global | NA |  |  |
| Gm0627_0302 | Regional | Calpain-2 |  | Protein binding^b^ (GO:0005515) |
| Gm0738_0160 | Global and regional | Complement factor B | Response to bacterium^a^ (GO:0009617) |  |
| Gm1108_0332 | Global and regional | Chitinase | Cell wall chitin metabolic process^b^ (GO:0006037), chitin catabolic process^b^ (GO:0006032),  digestion^b^ (GO:0007586), immune response^b^ (GO:0006955), response to acid^b^ (GO:0001101), response to fungus^b^ (GO:0009620) | Chitin binding^b^ (GO:0008061), chitinase activity^b^ (GO:0004568), lysozyme activity^b^ (GO:0003796), sugar binding^b^ (GO:0005529) |
| Gm1156_0573 | Global and regional | Enolase 1 | Negative regulation of cell growth^b^ (GO:0030308), negative regulation of transcription from RNA polymerase II promoter^b^ (GO:0000122) | Phosphopyruvate hydratase activity^b^ (GO:0004634), protein binding^b^ (GO:0005515), transcription corepressor activity^b^ (GO:0003714), transcription factor activity^b^ (GO:0003700) |
| Gm1386_0216 | Global and regional | Vitellogenin-2 | Response to estradiol stimulus^a^ (GO:0032355) |  |
| Aroma_1_9 | Regional | Aromatase | Response to estradiol stimulus^a^ (GO:0032355), response to xenobiotic stimulus^a^ (GO:0009410) |  |
| Hsp90 | Global and regional | Heat shock Protein 90 Beta | Negative regulation of proteasomal ubiquitin-dependent protein catabolic process^b^ (GO:0032435), positive regulation of nitric oxide biosynthetic process^b^ (GO:0045429), regulation of interferon-gamma-mediated signaling pathway^b^ (GO:0060334), regulation of type I interferon-mediated signaling pathway^b^ (GO:0060338), response to unfolded protein^b^ (GO:0006986) | Nitric-oxide synthase regulator activity^b^ (GO:0030235), TPR domain binding^b^ (GO:0030911) |
| Rhod_1_1 | Global and regional | Rhodopsin | Rhodopsin mediated phototransduction^a^ (GO:0009586) | Photoreceptor activity^a^ (GO:0009881), retinal binding^a^ (GO:0016918) |

**References**

Ashburner M, Ball CA, Blake JA, Botstein D, Butler H, Cherry JM, Davis AP, Dolinski K, Dwight SS, Eppig JT, Harris MA, Hill DP, Issel-Tarver L, Kasarskis A, Lewis S, Matese JC, Richardson JE, Ringwald M, Rubin GM, Sherlock G: **Gene Ontology: tool for the unification of biology**. *Nat Genet* 2000, **25**: 25-29.

Moen T, Hayes B, Nilsen F, Delghandi M, Fjalestad KT, Fevolden SE, Berg PR, Lien S: **Identification and characterisation of novel SNP markers in Atlantic cod: Evidence for directional selection.** *BMC Genet* 2008, **9**: 18.
